# Global transcriptomic analysis reveals Lnc-ADAMTS9 exerting an essential role in myogenesis through modulating the ERK signaling pathway

**DOI:** 10.1186/s40104-020-00524-4

**Published:** 2021-02-02

**Authors:** Liqi Wang, Ting He, Xin Zhang, Yubo Wang, Kai Qiu, Ning Jiao, Linjuan He, Jingdong Yin

**Affiliations:** State Key Laboratory of Animal Nutrition, College of Animal Science and Technology, China Agricultural University, No. 2 Yuanmingyuan West Road, Beijing, 100193 China

**Keywords:** Adipogenic precursors, LncRNA, Myogenic differentiation, Myogenic precursors, Myogenic proliferation, Pigs, Skeletal muscle

## Abstract

**Background:**

Long non-coding RNAs (lncRNAs) are emerging key regulators involved in a variety of biological processes such as cell differentiation and development. The balance between myogenesis and adipogenesis is crucial for skeletal muscle homeostasis in humans and meat quality in farm animals. The present study aimed to reveal the global transcriptomic profiles of adipogenic (Adi-) and myogenic (Myo-) precursors derived from porcine skeletal muscle and identify lncRNAs involved in the modulation of myogenesis homeostasis in porcine skeletal muscle.

**Results:**

In this study, a total of 655 novel individual lncRNAs including differentially expressed 24 lncRNAs, and 755 differentially expressed mRNAs were identified (fold change ≥2 or ≤ 0.5 and adjusted *P* < 0.05). Integrated results of Gene Ontology (GO) and Kyoto Encyclopedia of Genes and Genomes (KEGG) enrichment analysis accompanied by the variation of intracellular Ca^2+^ concentration highlighted Lnc-ADAMTS9 involved in the modulation of myogenesis homeostasis in porcine skeletal muscle. Although Lnc-ADAMTS9 knock-down did not alter the mRNA expression of *ADAMTS9,* we demonstrated that Lnc-ADAMTS9 can promote myogenic proliferation and myogenic differentiation of myogenic precursors through inhibiting the ERK/MAPK signaling pathway.

**Conclusion:**

We deciphered a comprehensive catalog of mRNAs and lncRNAs that might be involved in the regulation of myogenesis and adipogenesis homeostasis in the skeletal muscle of pigs. The Lnc-ADAMTS9 exerts an essential role in myogenesis through the ERK signaling pathway.

**Supplementary Information:**

The online version contains supplementary material available at 10.1186/s40104-020-00524-4.

## Introduction

The skeletal muscle makes up 20% – 50% of the total body mass in adult mammals and plays a pivotal role in regulating body metabolism and homeostasis [1]. The skeletal muscle not only implements the movement of bodies, but also serves as a tissue where ectopic lipid accumulated [2]. It is known that the adipocytes, myocytes, and fibroblasts all derive from mesenchymal progenitor cells during early embryonic development [3]. Myogenesis and adipogenesis occur competitively in the same microenvironment in skeletal muscle [4]. Furthermore, myogenesis is a crucial step for skeletal muscle development, regeneration, while excessive adipogenesis in skeletal muscle would suppress myogenesis and lead to myofibers infiltrated by lipids, and resultantly damages skeletal muscle contractive function and metabolism homeostasis [5]. In addition, the imbalance between myogenesis and adipogenesis in the skeletal muscle leads to various diseases including type-II diabetes, muscular atrophy, sarcopenia, and muscular dystrophy [6].

Myogenesis is regulated complicatedly by a cascade of intrinsic and extrinsic factors, including key transcriptional factors, miRNAs, and long non-coding RNAs (lncRNAs) [7], integrated by signaling pathways, such as Wnt/β-catenin [8], Notch [9], and MAPK [10] pathways. However, mechanisms that mediate the balance between myogenic and adipogenic differentiation remain unclear.

Accumulating evidence have shown that lncRNAs are key regulators involved in numerous important biological processes including stem cell maintenance and differentiation, myogenesis, and adipogenesis in particular [11]. Recently, numbers of lncRNAs have been characterized in myogenesis. For instance, LncMyoD, activated by *MyoD*, plays an important role in promoting myogenesis and skeletal muscle regeneration [7]. Lnc-mg is implicated in myogenesis and is required for MuSC differentiation by functioning as a competing endogenous RNA (ceRNA) of microRNA-125b in controlling IGF2 protein abundance [12]. In addition, Linc-RAM [13], MAR1 [14], and Neat1 [15] have been shown to play important roles in myogenesis, while a number of lncRNAs, such as MIR31HG [16] and TINCR [17], are implicated in adipogenesis. Therefore, the characterization of novel lncRNAs that mediate the balance between myogenesis and adipogenesis attracts intensive interest.

*Sus scrofa* provides a major source of animal-derived protein for humans as well as being an excellent model for understanding the balance between myogenesis and ectopic adipogenesis in the skeletal muscle, which determines muscle development potential and intramuscular fat accumulation in pigs [18]. Strengthening proliferation and differentiation potential of myogenic progenitor cells during fetal development promotes muscle growth and lean meat production while enhancing adipogenic commitment of adipogenic/fibrogenic progenitor cells in muscle improves marbling score and meat tenderness [19]. In this study, global transcriptional profiles of lncRNAs and mRNAs in myogenic and adipogenic precursor cells derived from porcine skeletal muscle were subjected to association analysis. Furthermore, a novel lncRNA XLOC_062039, located in the upstream of the *ADAMTS9* gene, named Lnc-ADAMTS9, was identified as a critical lncRNA, which promoted myogenic proliferation and differentiation through the ERK signaling.

## Materials and methods

### Cell isolation and culture

The preplate technique established by our lab [20], was applied to isolate adipogenic and myogenic precursors from the *longissimus dorsi* muscle of neonatal Yorkshire pigs of 3-day-old from different litters (*n* = 3), purchased from Beijing Pig Breeding Center, Beijing, China. Briefly, after pigs being humanely killed, 1 g of muscle was sampled from the *longissimus dorsi* muscle, minced and digested in 0.17% protease (Sigma, P8811, MO, USA) and 0.15% collagenase-type XI (Sigma, C9407, MO, USA) solution for 1 h, respectively. After filtered, centrifugated, and resuspended, the cell suspension was cultured in growth medium (GM) on a collagen I-coated dish at 37 °C and 5% CO_2_. GM containing DMEM/F12 (Hyclone, UT, USA) complemented with 10% FBS (Gibco, 10099-141, CA, USA), 2 mmol/L glutamine (Gibco, CA, USA), antibiotics (100 U/mL of penicillin and 100 mg/mL of streptomycin) and 5 ng/mL bFGF (basic fibroblast growth factor, PEPTECH, MA, USA). After 2 h, non-adherent cells were transferred to another dish and further collected after 72 h adhering. The first (0–2 h) and second (2–74 h) sets of adherent cells were adipogenic and myogenic precursors, respectively. The medium was changed every 2 days.

### Cell transfection and treatments

To explore the role of Lnc-ADAMTS9 in cell proliferation, myogenic precursors were plated on collagen I-coated six-well plates and transfected with 100 nmol/L scrambled siRNA or Lnc-ADAMTS9 siRNA (Ibsbio, Shanghai, China) using Lipofectamine 3000 (Invitrogen, Carlsbad, CA, USA) according to the manufacturer’s protocol. After 48 h of transfection, cells were collected or induced for myogenic differentiation. Lnc-ADAMTS9 siRNA sequences were listed as follows: sense 5′-GCAAAUGUAUCAACGGGAUUU-3′, antisense 5′-AUCCCGUUGAUACAUUUGCUU-3′; The sequence of the scrambled siRNA was 5′- UUCUCCGAACGUGUCACGUTT-3′ (sense strand) and 5′-ACGUGACACGUUCGGAGAATT-3′ (antisense strand).

U0126 (MedChem Express, HY-12031, Monmouth Junction, USA) was used as an ERK inhibitor [21] that depressed the phosphorylation of ERK1/2. To explore the role of Lnc-ADAMTS9 in myogenic differentiation, myogenic precursors were treated with scrambled siRNA + DMSO (Sigma, D2650, MO, USA), siLnc-ADAMTS9 + DMSO, and siLnc-ADAMTS9 + ERK inhibitor U0126 (5 μmol/L), respectively.

### Adipogenic and myogenic differentiation

To evaluate the differential potentials of adipogenic and myogenic precursors, cells were subjected to adipogenic and myogenic induction, respectively. During adipogenic induction, precursor cells were treated with adipogenic differentiation medium (DM), including 10% (v/v) FBS in DMEM, l μmol/L dexamethasone (Sigma, D4902, MO, USA), 0.5 mmol/L 1-methyl-3-isobutylmethyl-xanthine (Sigma, I5879, MO, USA), and 10 μg/mL insulin (Sigma, I6634, MO, USA). After 3 d, the DM was replaced by the maintenance medium (DMEM complemented with 10% FBS, 10 μg/mL insulin) for another 6 d. As for myogenic induction, precursor cells at 80–90% confluence were switched to myogenic differentiation medium consisting of 2% heat-inactivated horse serum (Thermo Fisher, 16050130, DE, USA) in DMEM for 4 d. Myogenic differentiation was assessed by the differentiation index and fusion index as described previously [22]. Differentiation index was calculated as the percentage of nuclei in myosin^+^ cells. Fusion index was calculated as the percentage of nuclei contained in myotubes (myosin^+^ cells with at least two nuclei).

### Oil red O staining

Lipid droplets were stained with Oil Red O to assess lipid accumulation and the efficiency of adipogenic differentiation. After the culture medium had been discarded, the cells were washed with PBS and H_2_O in turn. Subsequently, cells were fixed with 4% paraformaldehyde (PFA) for 30 min at room temperature (RT) and then incubated with 60% isopropanol for 5 min, then removed from the isopropanol and shifted to the incubation with 2 mL of Oil Red O working solution / well for 30 min at room temperature. Finally, Oil Red O working solution was aspirated and the cells were washed with H_2_O before taking images. Oil Red O-stained lipids were eluted in 100% isopropanol, and the optical density (OD) was measured at 520 nm.

### Immunofluorescence

After myogenic induction, cells were fixed with 4% PFA for 30 min at RT, permeabilized with 0.2% Triton X-100 for 10 min, blocked in blocking solution containing 5% bovine serum albumin in PBS for 1.5 h and incubated with anti-MHC (MY32 clone, Sigma M4276, MO, USA) overnight at 4 °C. After incubated with secondary antibody conjugated to Alexa Fluor 594 (ZSGB-BIO, ZF-0513, Beijing, China) for 1 h at RT, cells were incubated with DAPI (Solarbio, C0065, Beijing, China) for 10 min. Fluorescence was visualized using an Olympus fluorescence microscope (Olympus, Tokyo, Japan).

### Library preparation and sequencing analysis

Total RNAs were extracted from cultured adipogenic precursors and myogenic precursors (from three neonatal pigs, respectively) using a HiPure Total RNA Mini Kit with DNA filter (Magen, Guangzhou, China). The quantity and quality of RNA were evaluated using a NanoDrop spectrophotometer (Thermo Scientific, Wilmington, DE, USA). Ribosomal RNA was removed from total RNA using the Ribo-zero rRNA Removal Kit (Illumina, San Diego, CA, USA). The sequencing libraries were constructed with the NEB Next® Ultra™ Directional RNA Library Prep Kit for Illumina® (NEB, Ipswich, MA, USA) following the manufacturer’s recommendations. Briefly, the obtained RNA was cut into short fragments and synthesized the first-strand cDNA using random hexamer primer. The second-strand cDNA was synthesized using DNA Polymerase I and RNase H, in which dNTPs with dTTP were replaced by dUTP in the reaction buffer. After the adenylation of the 3′ ends of DNA fragments, adaptor with hairpin loop structure was ligated to prepare for hybridisation. The library fragments were purified with AMPure XP system (Beckman Coulter, Beverly, MA, USA) for cDNA fragments selecting. The USER Enzyme (NEB, Ipswich, MA, USA) was used with size-selected, adaptor-ligated cDNA before PCR. Finally, products were purified and library quality was evaluated on the Agilent Bioanalyzer 2100 system (Agilent Technologies, Santa Clara, CA, USA). The libraries were sequenced on an Illumina HiseqX Ten platform (Illumina, San Diego, CA, USA) and 150-bp paired-end reads were generated.

### Raw data quality control and alignment

The clean reads were obtained by filtering out the reads that contain adaptor or over 10% poly-Ns and over 50% of bases with Phred scores < 5 from raw reads of fastq format. Moreover, Q20, Q30, and GC content of the clean reads were calculated. The reads that passed the quality control were mapped to the *Sus scrofa* genome (Sscrofa10.2) from Ensemble Sus using HISAT.

### Prediction of novel porcine lncRNAs

High-quality reads were reconstructed to transcripts with StringTie (v1.0.4), and replicated transcripts were removed by Cuffcompare (v2.1.1). Subsequently, there are the following steps to predict the new lncRNA. Firstly, removing the transcripts with a total length of less than 200 nt. Secondly, filtering the ‘background’ transcripts for which the maximal expression was less than 2.0 and those present in only one sample or the number of exons was 1.0. Thirdly, the transcripts that overlapped with known mRNAs or lncRNAs on the same strand were discarded. Finally, the remaining transcripts without protein-coding potential jointly predicted by lncRNA prediction software Coding Potential Calculator (CPC, v0.9-r2), Pfam-scan (Pfam), and Coding Non Coding Index (CNCI, v2.1) were classified as lncRNAs.

### Differentially expressed mRNAs and lncRNAs

The number of reads mapped to each gene was count by HRSeq v0.5.3. Reads per kilo bases per million reads (RPKM) were applied to quantify gene expression. The R package DESeq2 (v1.4.5) was employed to figure out the differentially expressed lncRNAs and mRNAs between adipogenic and myogenic precursors. The resulting *P*-values were adjusted for multiple testing using Benjamini and Hochberg’s methods for controlling the false discovery rate (FDR). Fold change ≥2 or ≤ 0.5 and FDR < 0.05 were set as the differentially expressed mRNAs or lncRNAs.

### Bioinformatics analysis

The R package goseq (v1.16.2) was applied to perform the gene ontology (GO) enrichment analysis, and GO terms with a corrected *P* ≥ 0.05 were excluded. For the Kyoto Encyclopedia of Genes and Genomes (KEGG) analysis, the differentially expressed genes (DEGs) were mapped directly to the KEGG database. Cutoff criteria were set with Benjamini-Hochberg false discovery rate *q* < 0.05 for KEGG enrichment analysis.

LncRNAs exert regulative roles in the expression of mRNAs through direct competition with miRNA for binding loci or interaction with miRNA, known as mechanism of ceRNAs. In the present study, the putative interactions of lncRNA-miRNA and miRNA-mRNA were predicted by miRanda. Based on the predicted miRNA-mRNA and miRNA-lncRNA regulatory pairs, a ceRNA network was established in which the lncRNAs and mRNAs interacted via shared miRNAs. Cytoscape (Cytoscape Consortium, v.3.5.1, San Diego, CA, USA) were used to summarize and visualize ceRNA results. In order to investigate the function of lncRNAs, we predicted the *cis* target genes of differentially expressed lncRNAs by sequence homology (blastn, E-value < 1.0E-10 and identity > 99 and matched length ≥ 20 bp) between gene and lncRNA pairs. Firstly, we chose coding genes locating in 10 – 100 kb upstream and downstream of lncRNAs. Subsequently, we calculated the Pearson correlation coefficient by Microsoft Excel (Redmond, WA, USA) between lncRNAs and corresponding genes. Finally, lncRNAs *cis* target genes were identified with the absolute value of the correlation coefficient more than 0.6.

### Quantitative real-time PCR analysis

Total RNA was reverse-transcribed into cDNA using a PrimeScript RT reagent Kit with gDNA Eraser (Takara, RR047A, Japan) according to the manufacturer’s instructions. SYBR Green based qPCR was performed in a qTOWER 2.2 thermocycler (Analytik Jena, Jena, Germany). All samples were measured in triplicate. The primers of selected genes were listed in Table S3. Glyceraldehyde 3-phosphate dehydrogenase (GAPDH) was selected as an internal control. Relative gene expression level was calculated by 2^−ΔΔCt^ method [23].

### Intracellular calcium concentration measurement

Intracellular Ca^2+^ concentration was measured by flow cytometry as described previously [24] with some modifications. In brief, cells were incubated in 5 μg/mL fluo-3 acetoxymethyl ester (Cayman Chemical, Ann Arbor, MI, USA) at 37 °C for 30 min (protected from light) and then washed 3 times with PBS (containing 1% FBS) and centrifuged at 1000 r/min for 5 min. Subsequently, cells were resuspended in 200 μL PBS (containing 1% FBS). The conditions of the flow cytometer (BD Biosciences) were set to an excitation wavelength of 488 nm and a fluorescence signal acquisition wavelength of 525 nm. After excluding debris and dead cells, 20,000 cells were collected in each sample collection gate and the average, and fluorescence intensity of the cells were calculated.

### Proliferation assay

Myogenic precursors were plated into 96-well plates at a density of 5 × 10^3^ cells. Cells at 70–80% confluence were incubated with 5-ethynyl-2′-deoxyuridine (EdU) for 2 h before staining. Cell proliferation was detected using Cell-Light EdU Cell Proliferation Detection Kit (Ribobio, C10310, Guangzhou, China) following the manufacturer’s protocol. The percentage of proliferative cells was determined by quantitation of EdU-positive cells using an Olympus fluorescent microscope (Olympus, Tokyo, Japan).

### Flow cytometric analysis of cell cycle

Cells cultured in six-well plates with 70–80% confluence were transfected for 48 h, then fixed in 75% ethanol overnight at − 20 °C. Subsequently, the fixed cells were stained with propidium iodide (50 μg/mL) containing 10 μg/mL RNase A (Solarbio, Beijing, China), and then incubated for 30 min at 37 °C in the dark. Flow cytometric analysis was performed with a flow cytometer (BD Biosciences) and data were processed by FlowJo 7.6 software.

### Western blotting

Total cell protein lysate was extracted with cell lysis buffer containing protease inhibitor and protein phosphatase inhibitor cocktail. The relative protein expression was analyzed by western blot. Approximately 60–100 μg of total cell lysate was loaded and GAPDH was run as a loading control. In brief, protein samples were resolved on 8–12% SDS-PAGE gels and transferred to polyvinylidene fluoride membranes (Millipore, KGaA, Darmstadt, Germany). Membrane was blocked in TBS containing 5% (w/v) skimmed milk powder or bovine serum albumin at RT for 1 h and then incubated against corresponding primary antibodies at 4 °C overnight include ERK (CST, 4695S, MA, USA), p-ERK (CST, 4370S, MA, USA) and GAPDH (CST, 5174S, MA, USA), respectively. Blots were developed using DyLight 800-labeled secondary antibodies, detected with the Odyssey Clx (4647 Superior Street, LI-COR Biotechnology, Lincoln, NE, USA) and quantified by ImageJ (1.52v, NIH, USA).

### Statistical analysis

For comparison, data on lncRNA and mRNA between adipogenic and myogenic precursors were determined by the paired Student’s *t-*test using SAS (version 9.2, NC, USA), and the comparison concerning cell proliferation and differentiation were determined by Student’s *t-*test as well. Data were presented as means ± SEM. GraphPad Prism (version 8, CA, USA) was applied to graph. *P* < 0.05 was considered significant.

## Results

### Characterization of adipogenic and myogenic precursors

**Fig. 1:**
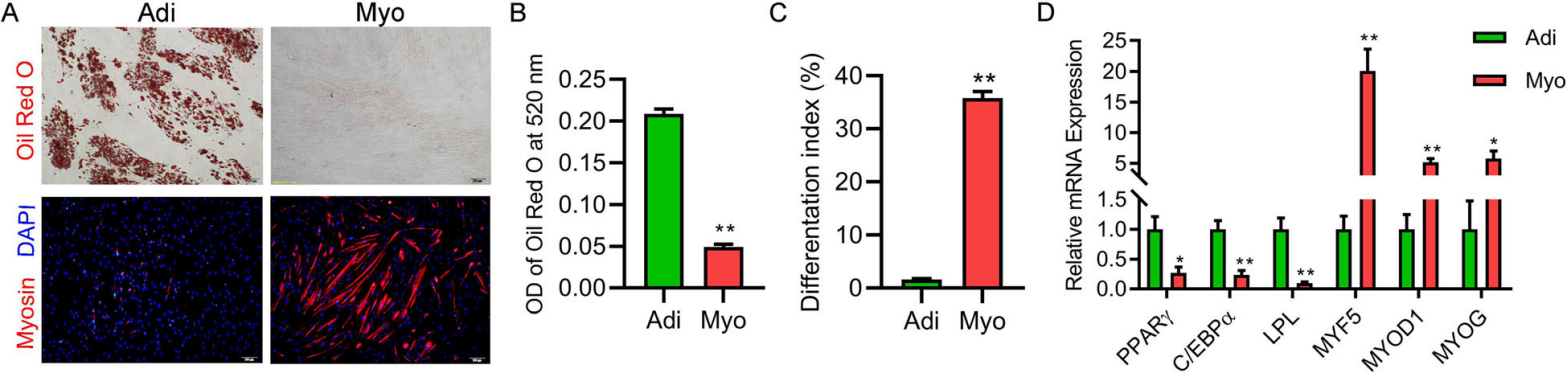
Characterization of adipogenic (Adi-) and myogenic (Myo-) precursors derived from the skeletal muscle of neonatal pigs (*n* = 3). **a** Oil Red O staining and immunofluorescence of adipogenic and myogenic precursors following 9-d adipogenic induction and 3-d myogenic induction, respectively. Oil Red O (red), Myosin (red), and DAPI (blue). **b** OD of Oil Red O at 520 nm and differentiation index between Adi and Myo. **c** Differentiation index between Adi and Myo. **d** Quantitative RT-PCR for the mRNA expression level of adipogenesis specific genes (*PPARγ, C/EBPα,* and *LPL*) and myogenesis specific genes (*MYF5, MYOD1,* and *MYOG*) following adipogenic or myogenic induction. Data are presented as mean ± SEM. Student’s *t*-test was used. Adi, adipogenic precursors; Myo, myogenic precursors. Scale bars, 100 μm. *P < 0.05, **P < 0.01 compared with adipogenic precursors

Upon adipogenic induction, adipogenic precursors differentiated into mature adipocytes, demonstrated by accumulating lipid droplets shown by Oil Red O staining (Fig. 1a, b). High mRNA expression levels of adipose-specific genes including peroxisome proliferator-activated receptor γ (*PPARγ*, *P =* 0.03), CCAAT-enhancer binding protein α (*CEBPα*, *P <* 0.01), and lipoprotein lipase (*LPL*, *P <* 0.01) were observed during the adipogenic differentiation of adipogenic precursors while little or no adipogenic potential was found in myogenic precursors (Fig. 1d).

Upon myogenic induction, myogenic precursors exhibited myogenic differentiation index (the proportion of myosin positive multi-nuclei myotubes), shown by immunofluorescence of myosin (Fig. 1a, c). A series of muscle-specific transcription factors, including myogenic factor 5 (*MYF5*, *P <* 0.01), myogenic differentiation 1 (*MYOD1*, *P <* 0.01), and myogenin (*MYOG*, *P =* 0.02), expressed more highly during the myogenic differentiation of myogenic precursors compared with those of adipogenic precursors (Fig. 1d). By contrast, adipogenic precursors exhibited very limited myogenic potential upon myogenic induction.

### Profile of mRNAs and lncRNAs in adipogenic and myogenic precursors

**Fig. 2:**
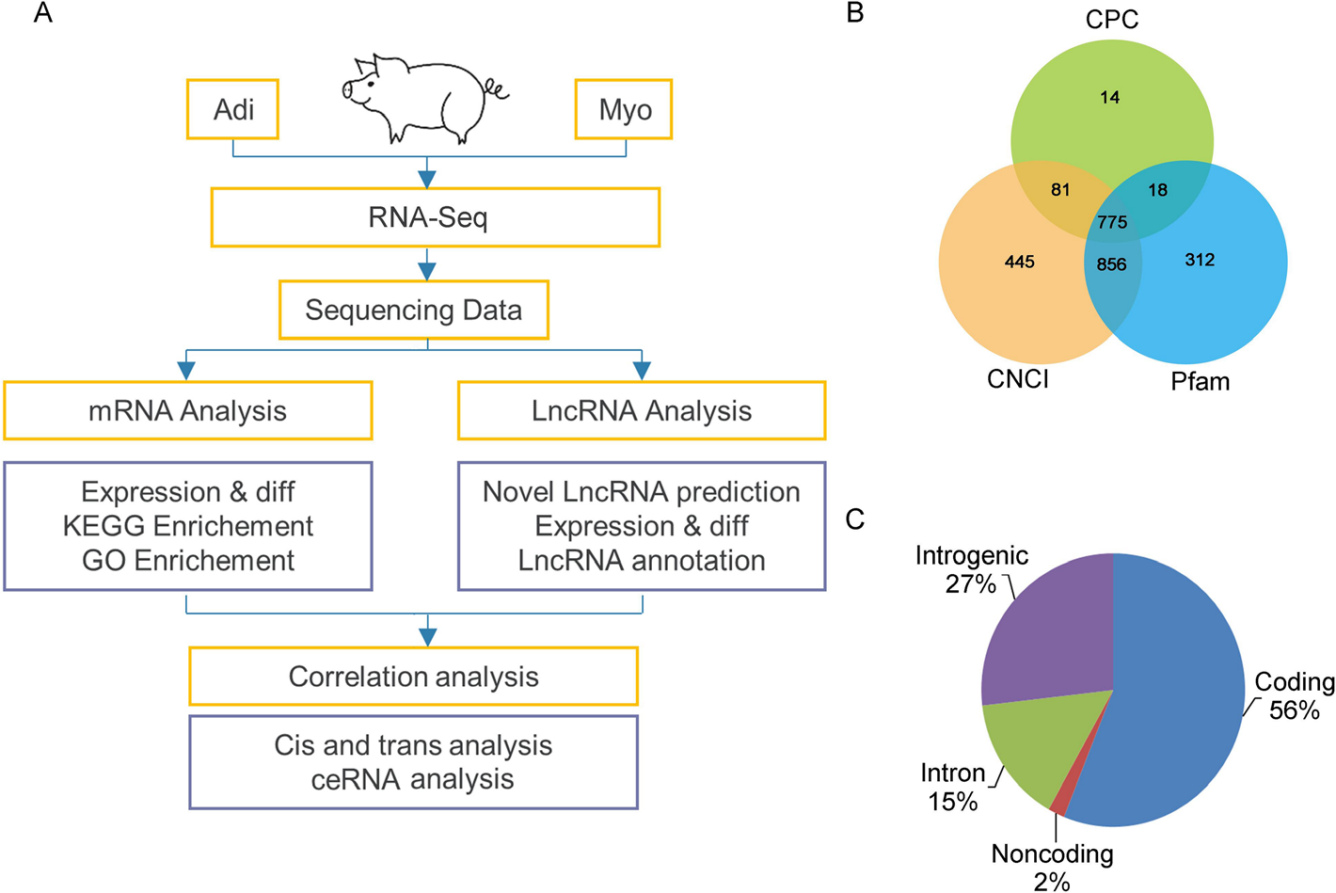
Identification of lncRNAs in adipogenic (Adi-) and myogenic (Myo-) precursors (*n* = 3) **a** Workflow of high-throughput RNA-seq of adipogenic and myogenic precursors. **b** Non-coding transcripts identified by three kinds of software (CPC, CNCI, Pfam) were statistically analyzed and drew into a Venn diagram. **c** Pie charts representing the percentage of nucleotide bases mapping to indicated genomic regions

Total RNA extracted from both adipogenic and myogenic precursors was subjected to high-throughput RNA-seq analysis following the workflow shown in Fig. 2a. We acquired 96 to 156 million and 89 to 111 million clean reads from the adipogenic and myogenic precursors, respectively (Table S1). In addition to 17,744 mRNAs identified, 775 putative long non-coding transcripts were identified (Fig. 2b), and among them, 27%, 15%, and 2% transcripts locate correspondingly in intergenic, intronic, and non-coding regions (Fig. 2c), representing 670 individual lncRNAs. After mapping to *Sus scrofa* genome from Ensembl, a total of 655 novel pig lncRNAs were discovered in the present study. The characteristics of lncRNA and mRNA concerning expression levels, exon numbers, open reading regions, sequence lengths, transcript numbers, and coding ability were shown in Fig. S1.

### Identification of differentially expressed mRNA and lncRNA

**Fig. 3:**
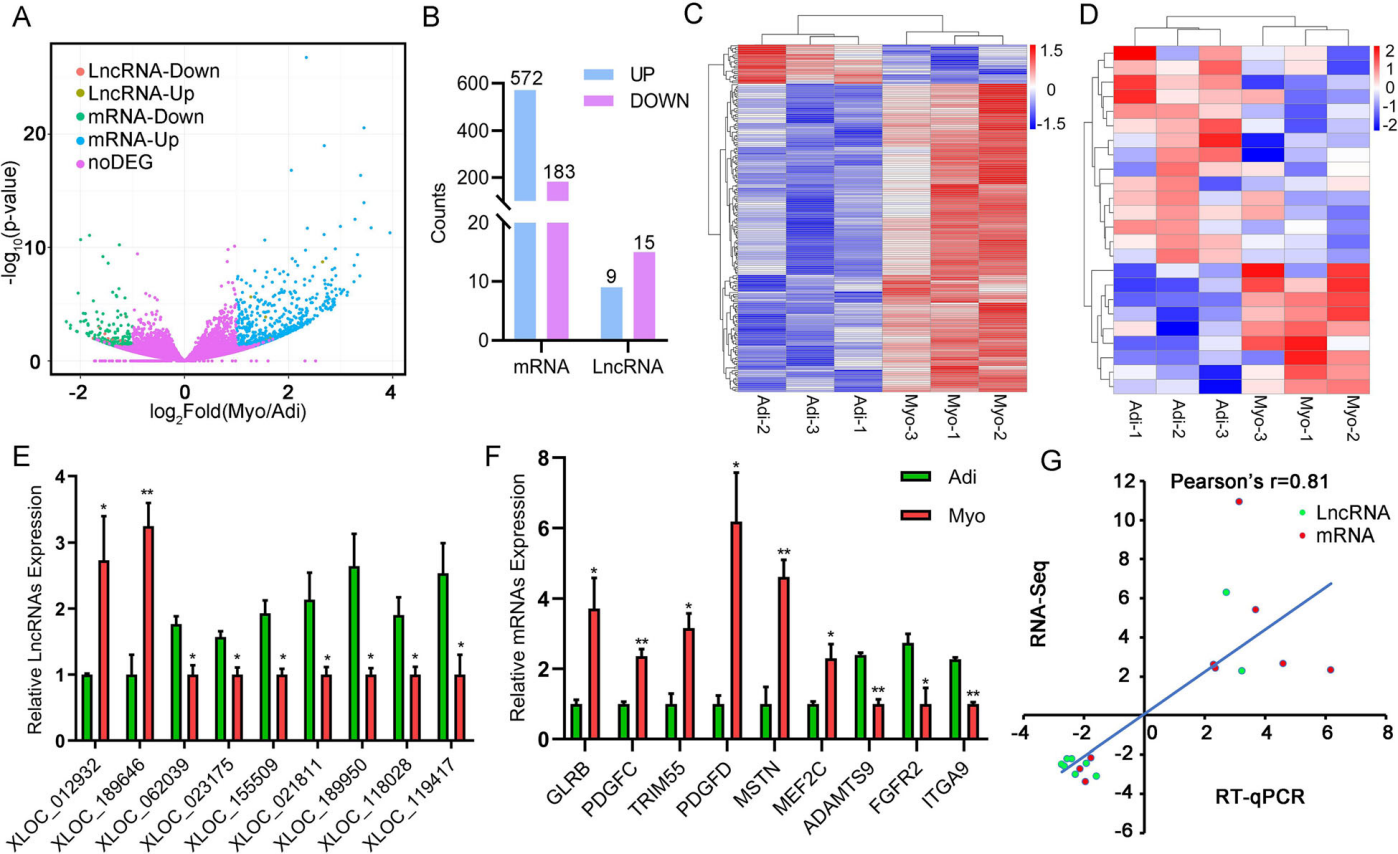
Differential expression levels of mRNAs and lncRNAs in adipogenic (Adi-) and myogenic (Myo-) precursors and validation of RNAseq results by qRT-PCR analysis. **a** Volcano map of all mRNAs and lncRNAs between Adi and Myo. **b** The up- and down-regulated number of mRNA and lncRNA between Adi and Myo. 572 up-regulated mRNAs, 183 down-regulated mRNAs, 9 up-regulated lncRNAs, and 15 down-regulated lncRNAs in myogenic precursors. Differentially expressed mRNAs (**c**) and lncRNAs (**d**) were clustered on the heat map by the normalized expression abundance. Relative lncRNA (**e**) and mRNA (**f**) expression were normalized to GAPDH. *n* = 4. Data are presented as mean ± SEM. **g** The correlation of fold change of differential expressed lncRNAs and mRNAs expression between the RNA-seq and RT-qPCR. A paired two-tailed Student’s *t*-test was used. * *P* < 0.05, ** *P* < 0.01

The distributions of differentially expressed mRNAs and lncRNAs between myogenic and adipogenic precursors were shown in Fig. 3a. A total of 755 differentially expressed genes (DEGs) at mRNA level (Table S3) and 24 differentially expressed lncRNAs were identified between adipogenic and myogenic precursors. Among them, 572 DEGs were up-regulated while 183 DEGs were down-regulated in myogenic precursors compared with those in adipogenic precursors. Meanwhile, 9 lncRNAs were up-regulated and 15 lncRNAs were down-regulated in myogenic precursors relative to adipogenic precursors (Fig. 3b; Table 1). Among these differentially expressed lncRNAs, XLOC_021529 and XLOC_001307 were the most up-regulated lncRNA while XLOC_155509 and XLOC_023175 were the most down-regulated lncRNA in myogenic precursors relative to adipogenic precursors. The heatmaps demonstrated the distinct expression pattern of the lncRNA and mRNA (Fig. 3c, d).

**Table 1:** Differentially expressed LncRNAs between in adipogenic and myogenic precursors

Gene ID	Fold change	*P-*value	Chromosome	Strand	Exon number	Exon sizes, bp
Up						
XLOC_021529	6.29	1.92E-09	Chr1	+	2	29; 202
XLOC_012932	2.42	2.37E-06	Chr9	–	1	1077
XLOC_079751	2.94	0.000,142	Chr14	–	2	190; 127
XLOC_090236	2.04	0.009,776	Chr15	+	2	17; 184
XLOC_001307	3.13	0.010,441	Chr7	+	3	156; 104; 1671
XLOC_164011	2.98	0.015,133	Chr6	+	1	259
XLOC_189646	2.28	0.025,817	Chr8	+	2	361; 114
XLOC_091324	2.70	0.028,678	Chr15	–	1	304
XLOC_165070	2.68	0.035,623	Chr6	–	2	21; 131
Down						
XLOC_062039	0.46	0.000,132	Chr13	+	2	592; 149
XLOC_023175	0.32	0.001,145	Chr1	–	2	229; 210
XLOC_118825	0.35	0.003,995	Chr2	–	2	164; 431
XLOC_155509	0.30	0.010,121	Chr5	–	2	1113; 182
XLOC_021811	0.36	0.010,917	Chr1	+	2	1110; 783
XLOC_189950	0.38	0.014,067	Chr8	+	3	114; 114; 295
XLOC_108084	0.42	0.016,205	Chr17	–	1	207
XLOC_189516	0.32	0.017,106	Chr8	+	2	52; 194
XLOC_190244	0.35	0.019,752	Chr8	–	2	10,531; 747
XLOC_118028	0.41	0.019,867	Chr2	+	2	20,801; 239
XLOC_119417	0.45	0.020,062	Chr2	–	3	1785; 113; 121
XLOC_154508	0.36	0.023,445	Chr5	+	2	427; 144
XLOC_155480	0.36	0.025,117	Chr5	–	2	221; 42
XLOC_178755	0.43	0.049,905	Chr7	+	2	662; 136
XLOC_190092	0.41	0.049,938	Chr8	–	2	1858; 188

### Quantitative real-time PCR verification

To validate the RNA-seq results, 9 differentially expressed lncRNAs (XLOC_012932, XLOC_189646, XLOC_062039, XLOC_023175, XLOC_155509, XLOC_021811, XLOC_189950, XLOC_118028 and XLOC_119417) and 9 differentially expressed mRNAs (*GLRB, PDGFC, TRIM55, PDGFD, MSTN, MEF2C, ADAMTS9, FGFR2* and *ITGA9*) were subjected to quantitative real-time PCR (Fig. 3e, f). The reliability of RNA-seq (transcriptome) analysis was validated by RT-qPCR (Pearson’s r = 0.81) (Fig. 3g).

### GO enrichment analysis

**Fig. 4:**
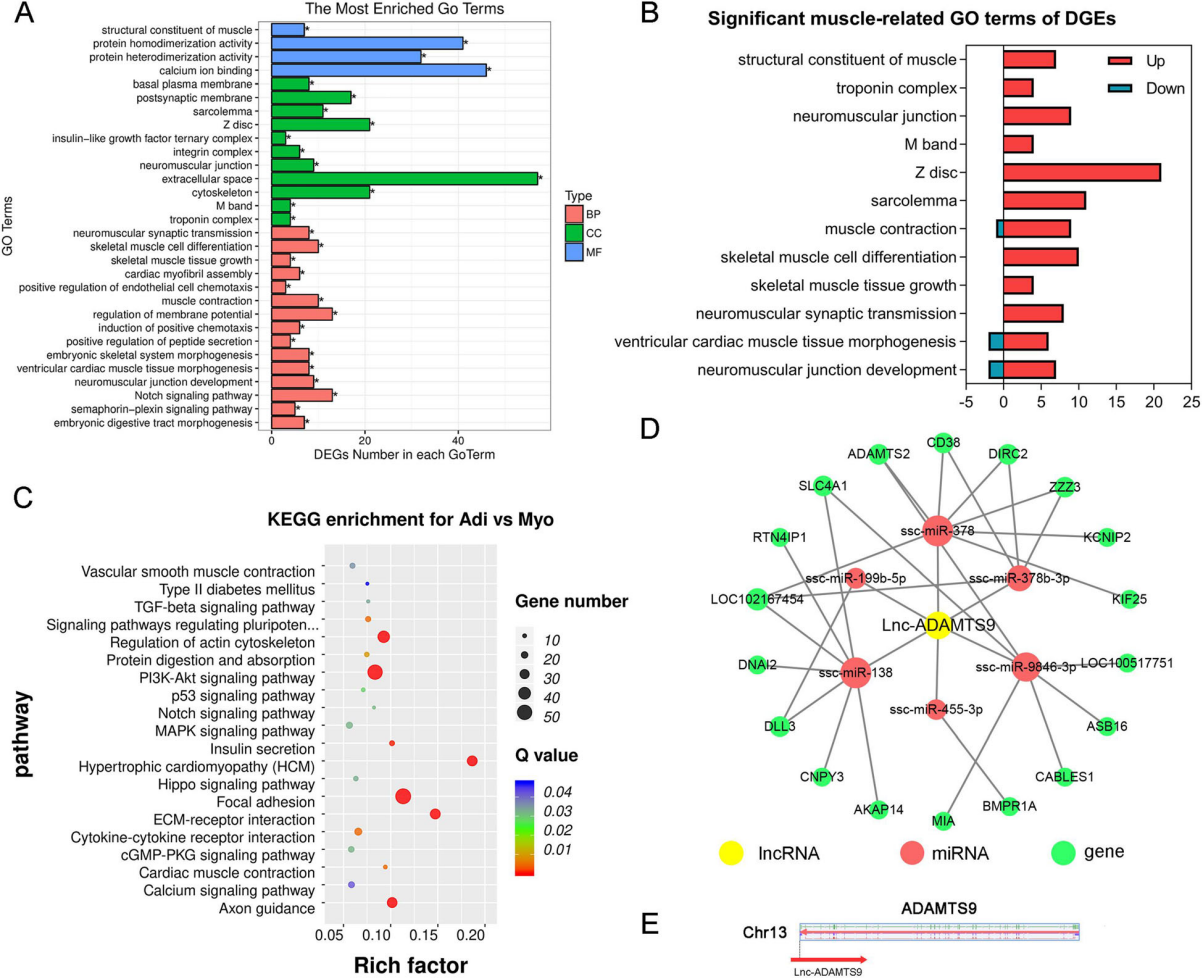
Bioinformatics including GO and KEGG enrichment analysis of DEGs, ceRNA network, and the schematic diagram of Lnc-ADAMTS9. **a** The most enrichment of GO terms of DEGs. **b** The significant muscle-related GO terms of DEGs. **c** The bubble chart of KEGG pathway enrichment between DEGs. **d** ceRNA network of Lnc-ADAMTS9. **e** The schematic diagram of porcine ADAMTS9 and Lnc-ADAMTS9

GO enrichment analysis showed that DEGs between adipogenic and myogenic precursors was annotated into 40 significant GO entries (*P* < 0.05), including 23 biological process (BP) GO entries, 13 cellular component (CC) GO entries, and 4 molecular function (MF) GO entries (Table S4). The top 30 significant GO terms (Fig. 4a) included calcium ion binding, extracellular space, the protein homodimerization activity, cytoskeleton, etc. Notably, GO entries related to muscle development, structure, and function, such as skeletal muscle tissue growth, structural constituent of muscle, and muscle contraction were significantly enriched (*P* < 0.05) (Fig. 4b). Among these terms, the majority of DEGs are up-regulated in myogenic precursors relative to adipogenic precursors.

### KEGG analysis

A total of 37 pathways were significantly enriched based on DEGs between adipogenic and myogenic precursors (Table S5). Among them, 20 significantly enriched pathways except those involved in human diseases were shown in Fig. 4c. In addition, Fig. S2 showed that DEGs, such as *FGFR2*, *ITGA6*, *ITGA9*, *PDGFC,* and *PDGFD*, acting as hubs to link important pathways including the Regulation of actin cytoskeleton, Focal adhesion, Calcium signaling pathway, MAPK signaling pathway, PI3K-Akt signaling pathway, cGMP-PKG signaling pathway, ECM-receptor interaction, Cytokine-cytokine receptor interaction and Signaling pathways regulating pluripotency of stem cells.

### Identification of candidate lncRNAs and putative ceRNA network

The target genes of lncRNAs were predicted to explore the potential function of lncRNAs. The *cis* target genes of differentially expressed lncRNAs were shown in Table 2. There were 17 differentially expressed lncRNAs with adjacent genes. Among these adjacent genes, only ENSSSCG00000011496 (*ADAMTS9*), the adjacent gene of the lncRNA XLOC_062039, is a DEG between adipogenic and myogenic precursors. *ADAMTS9* and lncRNA XLOC_062039, overlaps with a correlation coefficient of 0.98, both of them are down-regulated in myogenic precursors relative to adipogenic precursors. We named XLOC_062039 as Lnc-ADAMTS9. Based on the common target miRNAs, we constructed a putative ceRNA mechanism series connecting lncRNAs and mRNAs network about the Lnc-ADAMTS9 (Fig. 4d). We discovered that Lnc-ADAMTS9 is an antisense transcript locating on 5′ of coding DNA sequence (CDS) of *ADAMTS9* (Fig. 4e). A number of 6 miRNAs along with their linking 18 genes calculated in the present study should be explored in the future study.

**Table 2:** *Cis* target genes of differentially expressed lncRNAs

LncRNAs	LncRNA fold change	mRNA	Gene symbol	mRNA fold change	Relationship, bp	Correlation index
XLOC_021529	6.29	ENSSSCG00000005087	SIX 1	1.94	Downstream:23,747	0.96
XLOC_079751	2.94	ENSSSCG00000009880	SLC8B1	1.12	Overlap	0.61
XLOC_164011	2.98	ENSSSCG00000003102	PNMA8A	0.60	Upstream:22,659	−0.91
XLOC_189646	2.28	ENSSSCG00000021506	–	3.21	Downstream:72,987	0.88
XLOC_165070	2.68	ENSSSCG00000026699	ZNF180	1.06	Downstream:41,893	0.72
XLOC_062039	0.46	ENSSSCG00000011496	ADAMTS9	0.45	Overlap	0.98
XLOC_023175	0.32	ENSSSCG00000005751	COL5A1	0.75	Overlap	0.64
XLOC_155509	0.30	ENSSSCG00000000837	CHST11	0.98	Overlap	0.78
XLOC_021811	0.36	ENSSSCG00000005491	ATP6V1G1	1.08	Downstream:17,273	−0.88
		ENSSSCG00000005492	TMEM268	0.88	Overlap	0.76
XLOC_189950	0.38	ENSSSCG00000009230	WDFY3	0.71	Overlap	0.91
XLOC_108084	0.42	ENSSSCG00000007391	MATN4	1.67	Upstream:6245	−0.70
XLOC_189516	0.32	ENSSSCG00000008725	CYTL1	0.88	Downstream:15,131	0.90
XLOC_118028	0.41	ENSSSCG00000029992	NAV2	0.55	Overlap	0.98
XLOC_119417	0.45	ENSSSCG00000024043	ADAMTS2	0.80	Intron	0.83
XLOC_154508	0.36	ENSSSCG00000000191	KMT2D	0.56	Downstream:27,338	0.65
XLOC_155480	0.36	ENSSSCG00000030685	ARID2	0.77	Intron	0.64
XLOC_178755	0.43	ENSSSCG00000002526	RCOR1	0.67	Overlap	0.99

### The key role of Lnc-ADAMTS9 in myogenic proliferation and differentiation

**Fig. 5:**
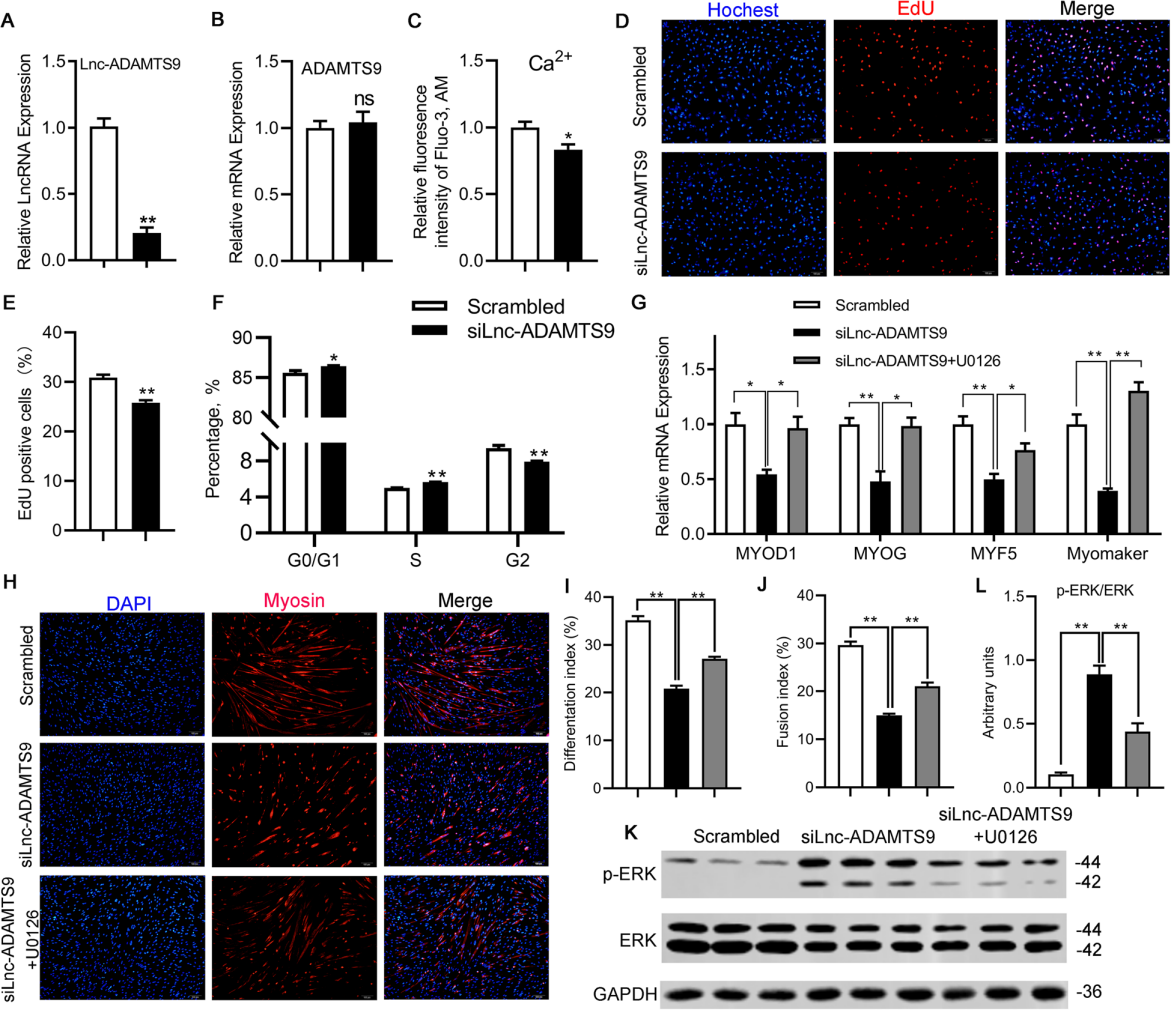
The role of Lnc-ADAMTS9 in proliferation and myogenic differentiation of myogenic precursors (*n* = 3) **a** The efficiency of knockdown by siLnc-ADAMTS9. **b** Quantitative RT-PCR for *ADAMTS9* mRNA expression level of myogenic precursors transfected with siLnc-ADAMTS9. **c** Quantitative result of intracellular Ca^2+^ signals of myogenic precursors transfected with siLnc-ADAMTS9. **d**, **e** EdU staining of myogenic precursors transfected with scrambled siRNA and siLnc-ADAMTS9. **f** Cell cycle analysis of myogenic precursors transfected with scrambled siRNA and siLnc-ADAMTS9. **g** The mRNA expression of genes involved in myoblast differentiation of myogenic precursors transfected with scrambled siRNA, siLnc-ADAMTS9, and siLnc-ADAMTS9 exposed to ERK inhibitor U0126 (5 μmol/L), respectively. **h** Representative immunofluorescence images of myogenic precursors transfected with scrambled siRNA, siLnc-ADAMTS9, and siLnc-ADAMTS9 exposed to ERK inhibitor U0126 (5 μmol/L), respectively. **i**, **j** Differentiation index and fusion index of myogenic precursors transfected with scrambled siRNA, siLnc-ADAMTS9, and siLnc-ADAMTS9 exposed to ERK inhibitor U0126 (5 μmol/L), respectively. **k**, **l** p-ERK (Thr202/Tyr204) protein levels of myogenic precursors transfected with scrambled siRNA, siLnc-ADAMTS9, and siLnc-ADAMTS9 exposed to ERK inhibitor U0126 (5 μmol/L) in DM, respectively. GAPDH was used as a loading control. Data are presented as mean ± SEM. An unpaired Student’s t-test was used. Scale bars, 100 μm. *P < 0.05, **P < 0.01

To identify the role of Lnc-ADAMTS9 in myogenesis, we firstly investigated the role of Lnc-ADAMTS9 on myogenic proliferation. Lnc-ADAMTS9 knockdown in myogenic precursors (*P <* 0.01, Fig. 5a) did not alter the mRNA expression level of ADAMTS9, the *cis* target gene of Lnc-ADAMTS9 (Fig. 5b). Furthermore, siLnc-ADAMTS9 decreased intracellular Ca^2+^ concentration compared with the scrambled (*P =* 0.04, Fig. 5c). Meanwhile, the transfection of siLnc-ADAMTS9 significantly slowed cell proliferation (*P <* 0.01, Fig. 5d, e). Flow cytometry analysis demonstrated that knockdown of Lnc-ADAMTS9 increased cell populations in the G_0_/G_1_ (*P =* 0.03) and S phase (*P <* 0.01), and decreased cell population in the G_2_ phase, resulting in arrested cells in the G_2_ phase (*P <* 0.01, Fig. 5f).

Besides, we also detected the role of Lnc-ADAMTS9 in myogenic differentiation. As a result, the knockdown of Lnc-ADAMTS9 remarkably decreased the mRNA expression of myoblast differentiation-specific genes including *MYOD1* (*P =* 0.01), *MYOG* (*P <* 0.01), *MYF5* (*P <* 0.01), and *Myomaker* (*P <* 0.01, Fig. 5g) compared with the scrambled. Meanwhile, the knockdown of Lnc-ADAMTS9 significantly blocked myotube formation (Fig. 5h), and depressed the differentiation index and fusion index compared with scrambled (*P <* 0.01, Fig. 5i, j). Particularly, we observed that the knockdown of Lnc-ADAMTS9 significantly enhanced the phosphorylation of ERK1/2 during myogenic differentiation (*P <* 0.01, Fig. 5k, l). In order to investigate the role of the ERK signaling pathway in Lnc-ADAMTS9 mediated-myogenic differentiation, U0126, an ERK inhibitor, was employed to treat siLnc-ADAMTS9 cells. We observed that U0126 treatment significantly promoted myotube formation, and increased the differentiation index and fusion index compared with those of Lnc-ADAMTS9-knockdown cells (*P <* 0.01, Fig. 5h, i, j). Meanwhile, the mRNA expression levels of genes involved in myoblast differentiation were also significantly increased upon U0126 treatment relative to that in siLnc-ADAMTS9 transfected myogenic precursors, whereas the ratio of p-ERK/ERK was significantly lowered (*P <* 0.01, Fig. 5k, l). Thus, Lnc-ADAMTS9 was required for myogenic proliferation and differentiation, and it can promote myogenic differentiation by inhibiting the ERK signaling pathway.

## Discussion

Previous studies have demonstrated that lncRNAs take part in the modulation of skeletal muscle cell differentiation and muscle development in humans and mice [25]. For example, SYISL promotes myoblast proliferation and myotube fusion but inhibits myogenic differentiation through interacting directly with PRC2 [26]; IncRNA YY1 regulates skeletal muscle regeneration through modulating both mitochondrial functions and glycolytic pathways in satellite cells [27]. Although some lncRNAs were identified in pigs [28], databases concerning pig lncRNA information are very limited, and only a few lncRNAs whose functions had been revealed, for example, lnc_000414 was characterized closely related to adipogenesis and function as an inhibitor in the proliferation of intermyocellular preadipocytes [29]. In the present study, we investigated the roles of lncRNAs in the modulation of myogenesis and adipogenesis homeostasis in skeletal muscle by comparing global transcriptomic landmark between myogenic and adipogenic precursors derived from the skeletal muscle of pigs.

Firstly, we found that 755 DEGs between myogenic and adipogenic precursors, sharing 257 common DEGs with our previous study [20], and most DEGs were up-regulated in myogenic precursors as well. Consistently, even more GO items were enriched in this study, the GO items concerning muscle constituents and functions, such as Z disc, M band, costamere, sarcolemma, basal plasma membrane, structural constituent of muscle, skeletal muscle contraction, and muscle filament sliding were similar to our previous study. Regarding cell differentiation and fate determination, GO items including skeletal muscle cell differentiation and embryonic skeletal system morphogenesis were also annotated by DEGs in this study. Accordingly, some similar GO items concerning cell components of extracellular matrix and cell adhesion, such as ECM-receptor interaction, cytoskeleton, calcium ion binding, and protein homodimerization/heterodimerization activity were also enriched. Notably, we presently annotated GO items including neuromuscular junction development and neuromuscular synaptic transmission, implying that neuromuscular junction exerts a critical role in the balance between myogenesis and adipogenesis in skeletal muscle, which warrants continual study in the future.

Aside from enriched pathways shown by our previous study, in the present study, KEGG analysis showed some pathways were implicated with both adipogenesis and myogenesis, such as focal adhesion, ECM-receptor interaction, PI3K-Akt signaling pathway, regulation of actin cytoskeleton, MAPK signaling pathway, and calcium signaling pathway. Studies have shown that focal adhesion protein levels are inversely related to embryonic stem cell adipogenic capacity, and increased levels of focal adhesion proteins inhibit lipogenesis in embryonic stem cells [30]. Cytoskeleton mechanics plays an important role in the balance of myogenic and adipogenic differentiation [31]. Hub genes involved in these pathways might be implicated in the distinct potential of adipogenic- and myogenic precursors. For example, FGFR4 can attenuate satellite cell differentiation during postnatal development [32] and its deficiency may improve glucose metabolism and insulin sensitivity in mice [33].

Ca^2+^ is necessary for cell proliferation and G_1_ is the most sensitive phase of the cell cycle to Ca^2+^ depletion [34]. Intracellular Ca^2+^ concentrations and calcium signaling pathways are important factors in regulating cell proliferation and differentiation [35, 36]. Intracellular Ca^2+^ activates the MAPK pathway and resultantly participates in stem cell differentiation into lineages [37, 38]. Consistently, we had evidence that intracellular Ca^2+^ and the calcium signaling pathway, as well as the MAPK signaling pathway, are advantageous for Myo-lineage cells to keep a potent differentiation potential [20, 24, 39].

As well known, the mammalian family of MAPK includes ERK, p38, and JNK. It had been indicated that ERK dimerizes in response to phosphorylation [40]. Preventing the dimerization of ERK diminishes the ERK cytoplasmic signaling [41]. ERK dimers are mainly detected in the cytoplasm and are related to scaffold proteins that serve as platforms of ERK dimerization, through which ERK binds to cytoplasmic substrates [42]. In addition to significantly enriched GO term protein homodimerization/heterodimerization activity, we also found that seven key DEGs between myogenic precursors and adipogenic precursors, including *PDE6C*, *CCL21*, *FGFR2*, *FGFR4*, *ALOX15*, *ADCYAP1,* and *FGF10*, positively regulate ERK1 and ERK2 cascades, implying that the ERK/MAPK pathway may be a powerful candidate mechanism underlying the modulation of myogenesis and adipogenesis homeostasis in porcine skeletal muscle.

It had been supposed that lncRNA probably expressed parallel to the adjacent mRNAs on the same chain [43]. Previous studies had suggested that lncRNAs modulated the expression of adjacent genes in a *trans-* and/or *cis*-acting way [44]. In the present study, we identified that two lncRNAs putatively targeted ADAMTS9 and SIX1, respectively, implicated in the determination of the distinct differential potential of adipogenic- and myogenic precursors. SIX1 has been considered as the main determinant of fast-twitch fiber type acquisition and maintenance [45] and binds to adipogenic and brown adipose tissue marker genes during adipogenesis [46]. Furthermore, lncRNA-Six1 can regulate SIX1 expression in a ceRNA-dependent way [47] or encodes a micropeptide to activate the SIX1 gene [48].

*ADAMTS9*, locating in cell surface/pericellular matrix proteolysis in diverse contexts, is a highly conserved secreted protease that remodels provisional ECM in morphogenetic processes [49] and takes part in the regulation of skeletal muscle insulin sensitivity [50]. In humans, ADAMTS9-AS2 was identified as an antisense transcript of the *ADAMTS9* [51]. ADAMTS9-AS2 inhibits cell proliferation via the miR-27a-3p/FOXO1 axis in clear cell renal cell carcinoma [52] and controls the chondrogenic differentiation in human mesenchymal stem cells through a ceRNA mechanism [53]. In the present study, we originally speculated that Lnc-ADAMTS9 may function through *ADAMTS9* in a lncRNA/mRNA pair manner. However, *ADAMTS9* mRNA expression was not altered by the knocked down of Lnc-ADAMTS9.

To investigate the role of Lnc-ADAMTS9 in proliferation and differentiation of myogenic precursors, we detected the effect of knockdown of Lnc-ADAMTS9 on proliferation and differentiation of myogenic precursors. We found that Lnc-ADAMTS9 knockdown decreased intracellular Ca^2+^ concentration. Ca^2+^ serves as a second messenger. Increased intracellular Ca^2+^ had been shown to play a critical role not only in myogenic differentiation but also in a variety of cellular and physiological functions like cell cycle progression and apoptosis [54]. Therefore, we deduced that Lnc-ADAMTS9 may up-regulate intracellular Ca^2+^ and promote myogenesis through cooperating with Ca^2+^, which warrants further validation. Meanwhile, in the present study, we observed that LncRNA-ADAMTS9 knockdown reduced the proliferation of myogenic precursors accompanied by the increased cell population in the G_1_ and S phase and decreased cell population in the G_2_ phase, indicating that Lnc-ADAMTS9 plays a key role in promoting myogenic precursor proliferation.

Besides, ERK1/2 cascade plays an opposite role in proliferation and myogenic differentiation by suppressing the expression of muscle-specific genes [55]. The controversy exists on the role of ERK in myogenic differentiation. For example, FGF13 activated the ERK1/2 pathway to down-regulate Spry1 protein expression and lead to C2C12 cell differentiation inhibition [56]. However, EPHA2 was required for myogenic differentiation, and it may promote myogenic differentiation through ERK signaling [57]. In the present study, we observed that Lnc-ADAMTS9 knockdown activated the ERK1/2 pathway, while depressed myogenic differentiation and impaired myotube formation. Moreover, ERK inhibitor U0126 can recover the impaired myogenic differentiation potential of myogenic precursors induced by siLnc-ADAMTS9 knockdown. It strongly supported that Lnc-ADAMTS9 promoted myogenic differentiation through depressing the ERK1/2 pathway, and ERK1/2 negatively alter myogenic differentiation of myogenic precursors derived from the skeletal muscle of pigs.

Above all, it is conceivable that Lnc-ADAMTS9 serves as a positive role in promoting proliferation and myogenic differentiation of myogenic precursors through the regulation of the ERK1/2 pathway.

## Conclusions

We provided a comprehensive catalog of mRNAs and lncRNAs involved in the regulation of myogenesis and adipogenesis homeostasis in the skeletal muscle of pigs. A novel functional lncRNA, named Lnc-ADAMTS9, was identified and characterized to be implicated in the modulation of proliferation and myogenic differentiation of myogenic precursors. We proved that Lnc-ADAMTS9 is required for proliferation, myogenic differentiation of myogenic precursors through mediating the ERK signaling pathway. The role of Lnc-ADAMTS9 in adipogenesis warrants future study, which embodies the significance of intramuscular fat accumulation in pigs.

## Supplementary Information

**Additional file 1: Table S1.** Summary of reads mapping to the reference genome.

**Additional file 2: Table S2.** Primer sequences used in qRT-PCR analysis.

**Additional file 3: Figure S1.** Comparison of features of lncRNAs and mRNAs between myo- and adio-precursors. A) The comparison of lncRNA and mRNA expression levels. B) The comparison of lncRNA and mRNA exon number. C) The comparison between lncRNA and mRNA open reading region. D) Comparison of the length of lncRNA and mRNA transcripts. E) The comparison of the number of lncRNA and mRNA transcripts. F) The comparison of the coding ability scores of known lncRNA, mRNA, and new lncRNA.

**Additional file 4: Figure S2.** Network of hub DEGs in key KEGG pathways. Ovals represent hub DGEs and diamonds represent KEGG pathways. Networks were visualized by Cytoscape (v3.5.1).

**Additional file 5: Table S3.** List of 755 DEGs between adipogenic and myogenic precursors derived from pig skeletal muscle. **Table S4.** List of GO terms based on 755 DEGs between adipogenic and myogenic percursors derived from pig skeletal muscle. **Table S5.** List of KEGG terms based on 755 DEGs between adipogenic and myogenic percursors derived from pig skeletal muscle.

**Additional file 6: **Graphical abstract.

## Data Availability

All data generated or analyzed during this study are included in this published article [and its supplementary information files].

## Abbreviations

Adi: Adipogenic precursors
CDS: Coding DNA sequence
ceRNA: Competing endogenous RNA
DEG: Differentially expressed gene
DM: Differentiation medium
EdU: 5-ethynyl-2′-deoxyuridine
FDR: False discovery rate
GM: Growth medium
GO: Gene Ontology
KEGG: Kyoto Encyclopedia of Genes and Genomes
LncRNA: Long non-coding RNA
Myo: Myogenic precursors
OD: Optical density
PFA: Paraformaldehyde
RPKM: Reads per kilo bases per million reads
RT: Room temperature
