# Supplementary material for: Global transcriptomic analysis reveals Lnc-ADAMTS9 exerting an essential role in myogenesis through modulating the ERK signaling pathway
Source: J Anim Sci Biotechnol. 2021 Feb 2;12:4. doi: 10.1186/s40104-020-00524-4 (PMC7852153; doi:10.1186/s40104-020-00524-4)
Supplement: Supplementary file 1 — Additional file 1: Table S1. Summary of reads mapping to the reference genome. [file 40104_2020_524_MOESM1_ESM.docx]

**Table S1 Summary of reads mapping to the reference genome**

| Items | Adi-1 | Adi-2 | Adi-3 | Myo-1 | Myo-2 | Myo-3 |
| --- | --- | --- | --- | --- | --- | --- |
| Raw reads | 161,706,458 | 97,866,072 | 101,675,122 | 114,293,418 | 91,703,290 | 97,297,282 |
| Clean reads | 156,768,154 | 96,154,974 | 99,871,848 | 111,596,124 | 89,810,756 | 95,581,170 |
| Clean reads ratio, % | 96.95 | 98.25 | 98.23 | 97.64 | 97.94 | 98.24 |
| Proportion of Q30, % | 94.06 | 93.69 | 93.63 | 93.81 | 93.90 | 93.96 |
| Mapped reads, % | 79.49 | 81.95 | 82.23 | 79.98 | 81.57 | 81.63 |
| Unique mapped reads, % | 67.74 | 71.67 | 68.53 | 66.04 | 68.44 | 69.13 |
