# Supplementary material for: Global transcriptomic analysis reveals Lnc-ADAMTS9 exerting an essential role in myogenesis through modulating the ERK signaling pathway
Source: J Anim Sci Biotechnol. 2021 Feb 2;12:4. doi: 10.1186/s40104-020-00524-4 (PMC7852153; doi:10.1186/s40104-020-00524-4)
Supplement: Supplementary file 2 — Additional file 2: Table S2. Primer sequences used in qRT-PCR analysis. [file 40104_2020_524_MOESM2_ESM.docx]

**Table S2 Primer sequences used in qRT-PCR analysis**

| **Gene** | **Forward** | **Reverse** |
| --- | --- | --- |
| XLOC_012932 | CAGGGGCCTCCTTATTTGGAA | CTGCCCTCAGAGTGTTGACAT |
| XLOC_189646 | GGGAAACTGGTTCAGGTCCA | CGCGGGGTACTTTCCACATA |
| XLOC_062039 | ATCTGCCTTTCTTCCCAACTT | GGCCCTCCAGGAAGTTATATTCA |
| XLOC_023175 | GGGGGCCACGGATAATTTCA | ACCACGAGTCTGTCCTGTCT |
| XLOC_155509 | AGCTCTCCTCCATCTGGTGT | CCAGCAGAATGGGAGCAGAA |
| XLOC_021811 | GGCAGTGAAGATGAAGGCCT | AGGAACACTTGCACTGGCTT |
| XLOC_189950 | CGGTGGTTGGTTGTGGTTTC | ACCACTGACGGAACAGAACC |
| XLOC_118028 | GGCTGTCTTGGACCAGTCTC | GTCATGAAGACTGCCCAGCT |
| XLOC_119417 | GCTGCAAGAGACTCCCTGAG | TCGTTTCCGGATCTGTTGCA |
| *GAPDH* | TCGGAGTGAACGGATTTG | CCTGGAAGATGGTGATGG |
| *PPARγ* | GAGGGCGATCTTGACAGGAA | GCCACCTCTTTGCTCTGCTC |
| *C/EBPα* | GGCCAGCACACACACATTAGA | CCCCCAAAGAAGAGAACCAAG |
| *LPL* | AAGTATTGGCATCCAGAAACC | TTGATCTCATAGCCCAAGTTGT |
| *MYOD1* | GGTGACTCAGACGCATCCAG | AGGTGCCGTCGTAGCAGTTC |
| *MYOG* | CTGCTCACAGCTGACCCTAC | GGTTTCATCTGGGAAGGCCA |
| *MYF5* | AGTTCGGGGACGAGTTTGAG | TCAAACGCCTGGTTGACCTT |
| *Myomaker* | CAGCAGATGAAGGACCAACG | CGCAGTGGTAGAAGCTGTGG |
| *GLRB* | GAGTATGCTGTCGTCCAGGTGATG | AAGGTGCTGATGTGAACAGGAGTG |
| *PDGFC* | GTGTGACTGCGTGTGCAGAG | CCGAGATGGAGGATGGAGAT |
| *TRIM55* | AGCAAGAGTCCACCAGACCAGAG | TCGCAGTTCAGGCAGTAGATGTTG |
| *PDGFD* | TGACCTGGCGGCTCCATTCC | CTTCCTTGTGTCCACACCATCGTC |
| *MSTN* | ACTCGACTGTGATGAGCACTCAAC | ACACTCTCCAGAGCAGTAATTGGC |
| *MEF2C* | TTCCACCAGGCAGCAAGAATACG | GGAGTAGCCAATGACTGAGCAGAC |
| *ADAMTS9* | CGTGTCCGCTCTACACTTGG | GCCATGAACCTCACCACCTT |
| *FGFR2* | ACAACTCGCCTCTCCTCCACAG | CACCGCTTCAGCCATGACCAC |
| *ITGA9* | GAGCCTTCATGTCTGACAGTGTGG | ACTGAGGTGCTGTGATGTTGATGG |
