# Supplementary figures and images for: Global transcriptomic analysis reveals Lnc-ADAMTS9 exerting an essential role in myogenesis through modulating the ERK signaling pathway
Source: J Anim Sci Biotechnol. 2021 Feb 2;12:4. doi: 10.1186/s40104-020-00524-4 (PMC7852153; doi:10.1186/s40104-020-00524-4)

A

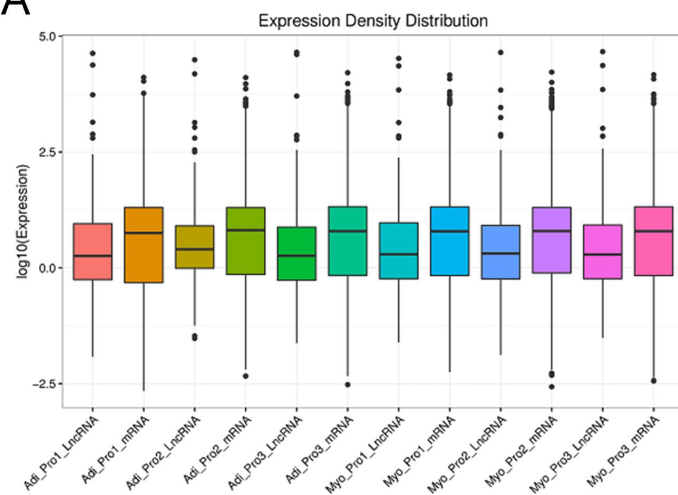

B

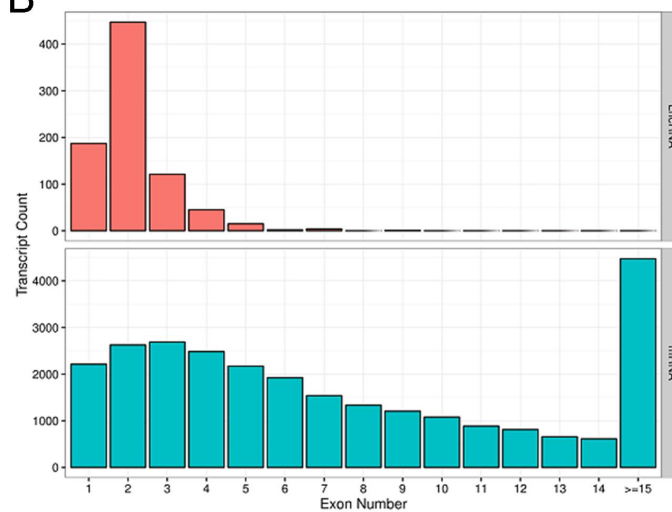

C

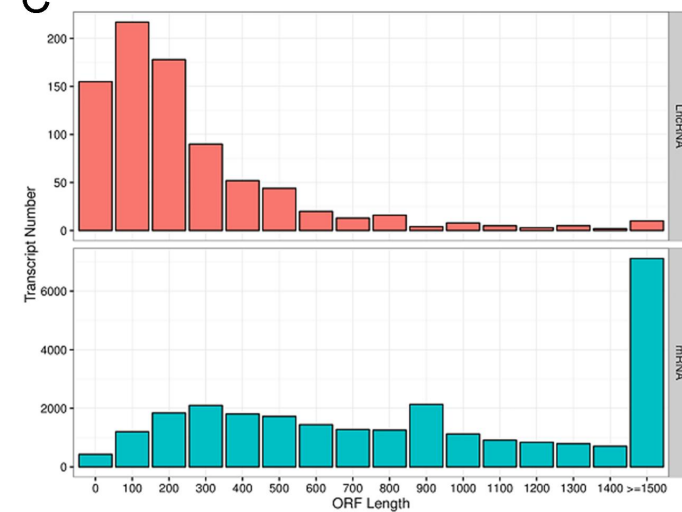

D

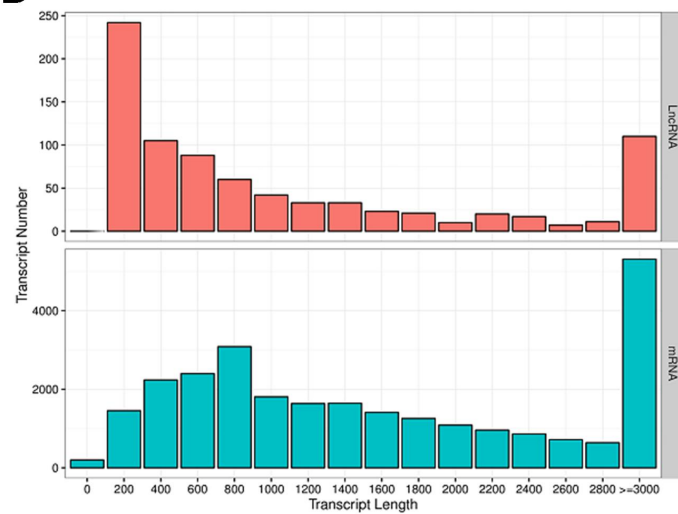

E

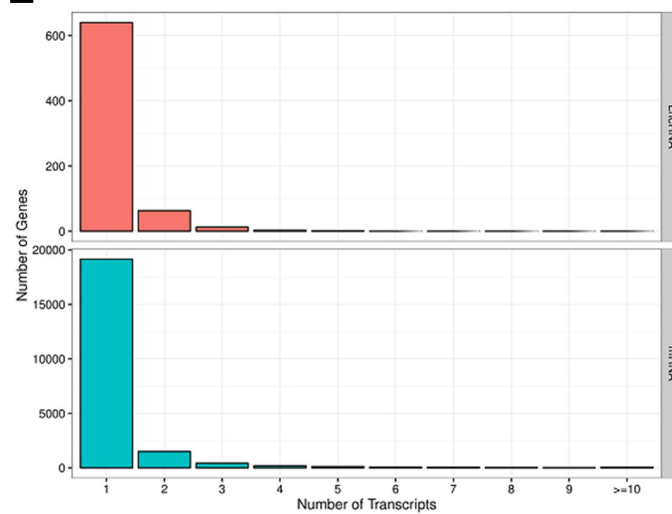

F

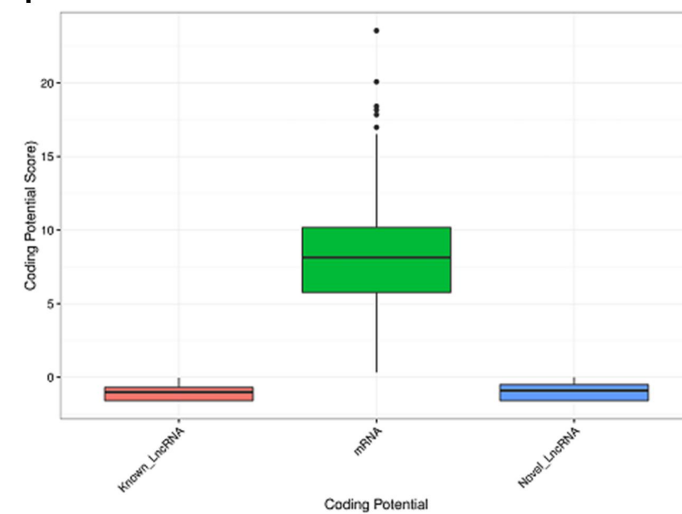

Supplement: Supplementary file 3 — Additional file 3: Figure S1. Comparison of features of lncRNAs and mRNAs between myo- and adio-precursors. A) The comparison of lncRNA and mRNA expression levels. B) The comparison of lncRNA and mRNA exon number. C) The comparison between lncRNA and mRNA open reading region. D) Comparison of the length of lncRNA and mRNA transcripts. E) The comparison of the number of lncRNA and mRNA transcripts. F) The comparison of the coding ability scores of known lncRNA, mRNA, and new lncRNA. [file 40104_2020_524_MOESM3_ESM.pdf]

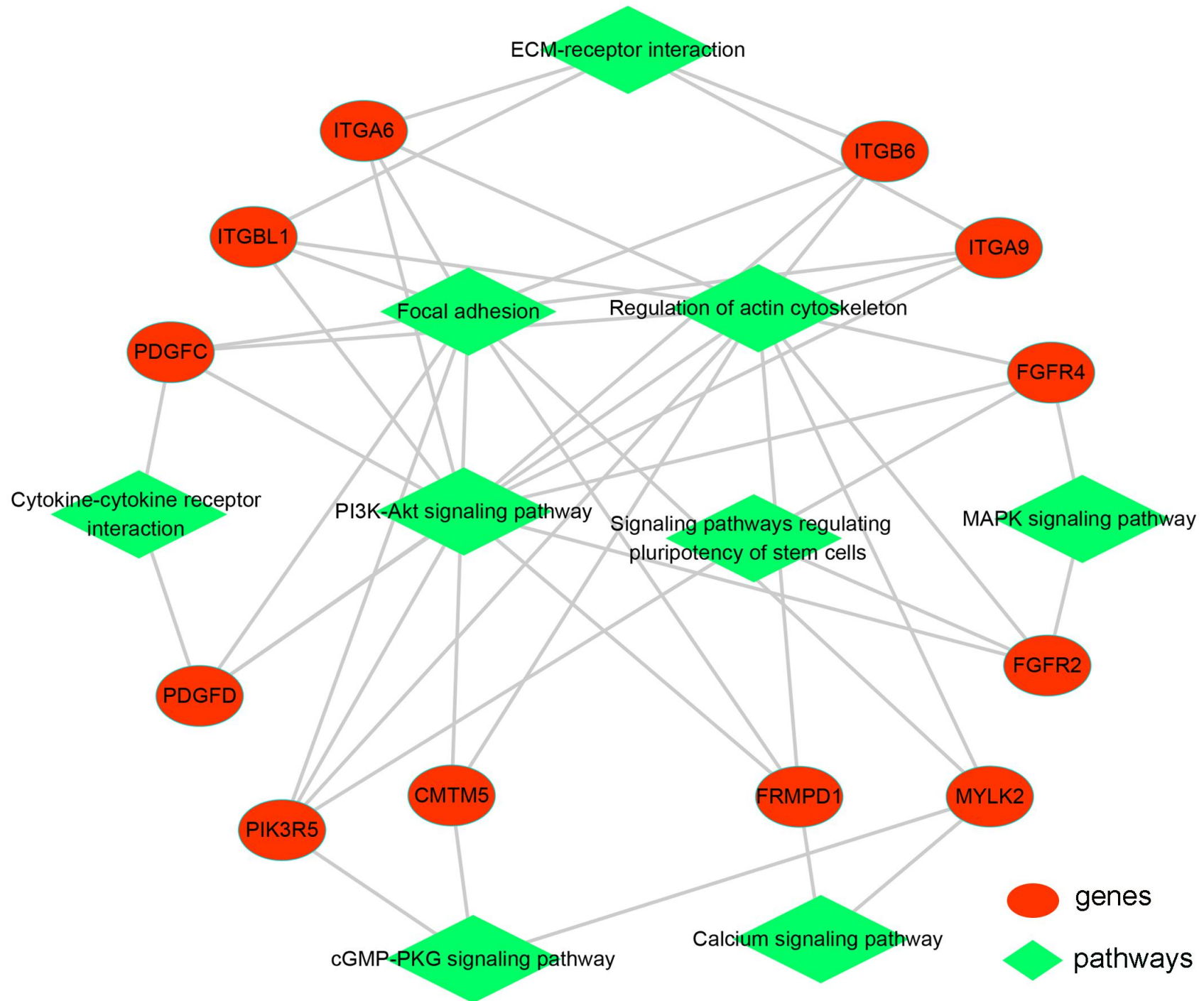

Supplement: Supplementary file 4 — Additional file 4: Figure S2. Network of hub DEGs in key KEGG pathways. Ovals represent hub DGEs and diamonds represent KEGG pathways. Networks were visualized by Cytoscape (v3.5.1). [file 40104_2020_524_MOESM4_ESM.pdf]
