# Supplementary material for: Global transcriptomic analysis reveals Lnc-ADAMTS9 exerting an essential role in myogenesis through modulating the ERK signaling pathway
Source: J Anim Sci Biotechnol. 2021 Feb 2;12:4. doi: 10.1186/s40104-020-00524-4 (PMC7852153; doi:10.1186/s40104-020-00524-4)
Supplement: Supplementary file 6 — Additional file 6: Graphical abstract. [file 40104_2020_524_MOESM6_ESM.pdf]

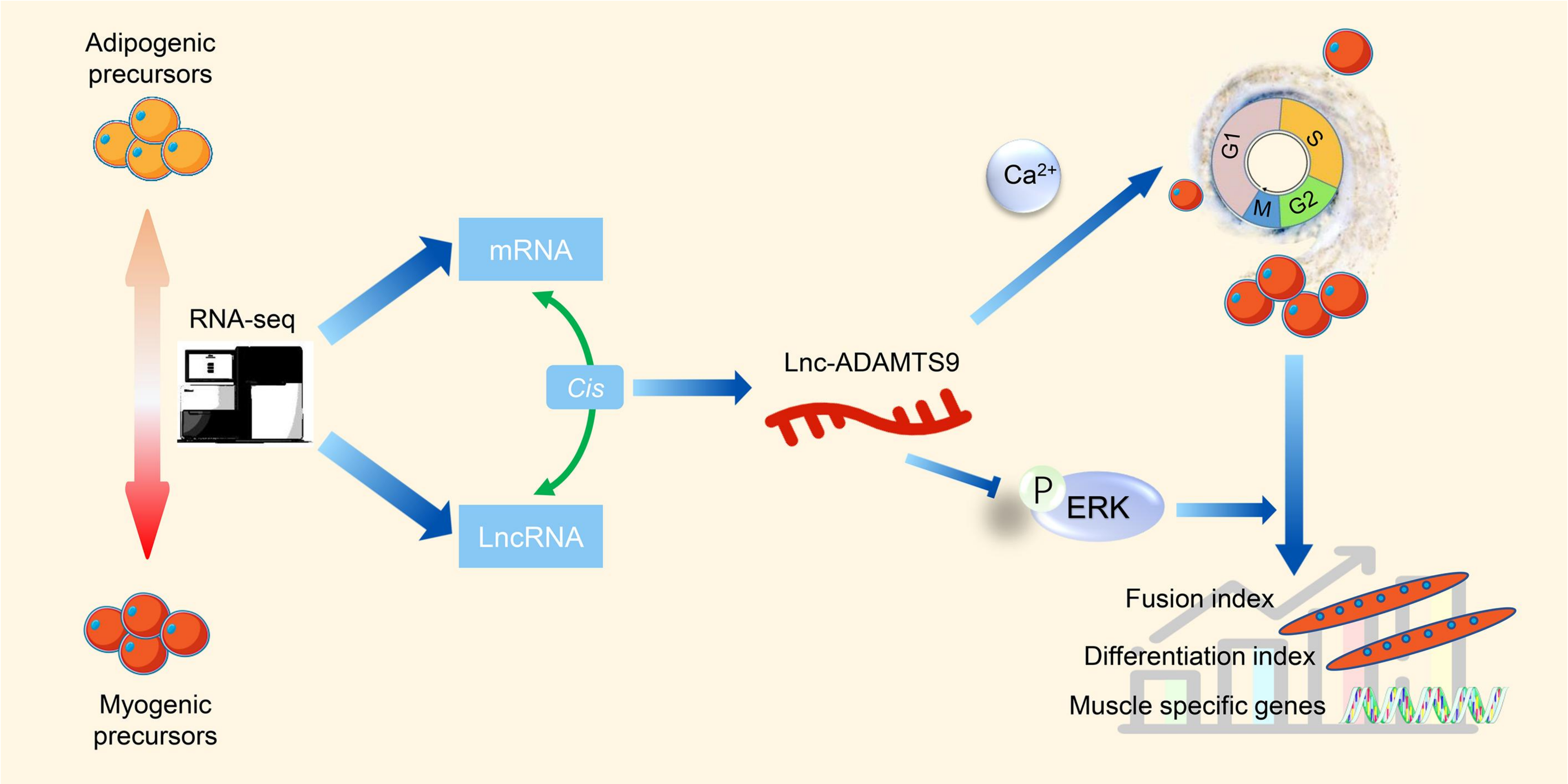
Adipogenic precursors
RNA-seq
mRNA
Cis
LncRNA
Myogenic precursors
Lnc-ADAMTS9
Ca2+
G1
S
M
G2
P
ERK
Fusion index
Differentiation index
Muscle specific genes
